# Deviant Behavior of Pedestrians: A Risk Gamble or Just Against Automated Vehicles? How About Social Control?

**DOI:** 10.3389/frobt.2022.885319

**Published:** 2022-07-08

**Authors:** Hatice Şahin, Sebastian Hemesath, Susanne Boll

**Affiliations:** ^1^ Media Informatics and Multimedia Systems Group, Department of Computing Science, University of Oldenburg, Oldenburg, Germany; ^2^ Institute for Social Sciences, University of Oldenburg, Oldenburg, Germany

**Keywords:** automated vehicles, self-driving cars, social control, deviant behavior, bullying, virtual reality, pedestrian, vulnerable road users

## Abstract

Recent evidence suggests that the assumed conflict-avoidant programming of autonomous vehicles will incentivize pedestrians to bully them. However, this frequent argument disregards the embedded nature of social interaction. Rule violations are socially sanctioned by different forms of social control, which could moderate the rational incentive to abuse risk-avoidant vehicles. Drawing on a gamified virtual reality (VR) experiment (*n* = 36) of urban traffic scenarios, we tested how vehicle type, different forms of social control, and monetary benefit of rule violations affect pedestrians’ decision to jaywalk. In a second step, we also tested whether differences in those effects exist when controlling for the risk of crashes in conventional vehicles. We find that individuals do indeed jaywalk more frequently when faced with an automated vehicle (AV), and this effect largely depends on the associated risk and not their automated nature. We further show that social control, especially in the form of formal traffic rules and norm enforcement, can reduce jaywalking behavior for any vehicle. Our study sheds light on the interaction dynamics between humans and AVs and how this is influenced by different forms of social control. It also contributes to the small gamification literature in this human–computer interaction.

## 1 Introduction

**FIGURE 1:**
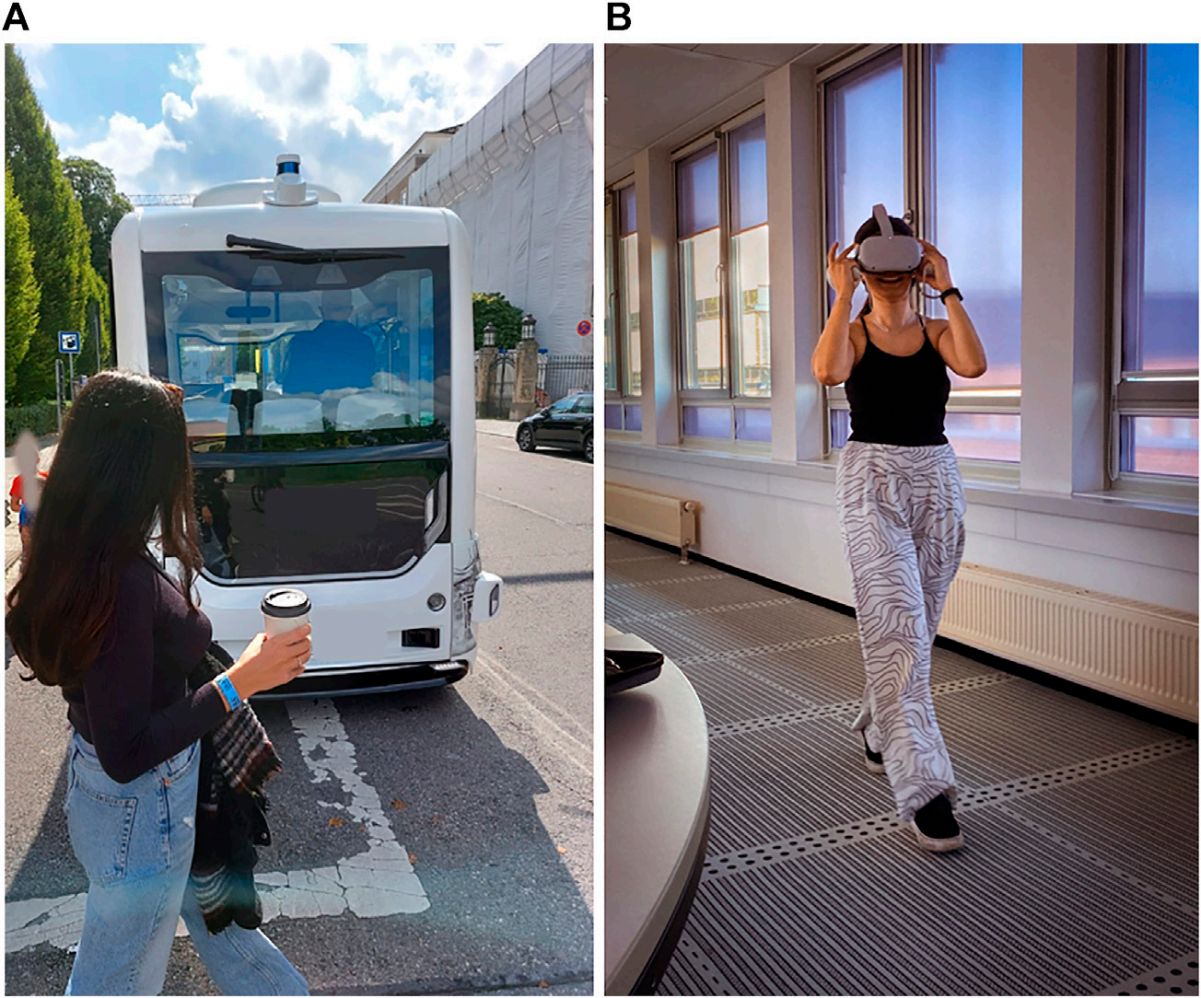
Human–AV interaction in everyday traffic and in pedestrian simulators. **(A)** In the future, pedestrians will interact with AVs with higher levels of automation daily. **(B)** VR enabled us to utilize our meeting room with a long corridor for a safe testing environment for our pedestrian simulator.

In the near future, we can expect mixed traffic mobility in which vehicles with no, partial, or full automation (SAE, 2016) will coexist and cooperate with human traffic participants, including vulnerable road users (VRUs) such as pedestrians and cyclists (Holländer et al., 2021) (see Figure 1A). At first sight, road traffic appears to be a highly regulated system in which agents act according to traffic code rather than their normative beliefs and values. Many interactions in urban traffic, however, are not only weakly regulated and observed; they also rely on established social norms and practices. Moreover, there might be many other factors in traffic that can shape the behavior of individuals. There are many traffic situations in which cooperative behavior is exercised, such as letting a pedestrian pass in slow-flowing traffic on an urban street even though there is no traffic light or pedestrian crossing. In such situations, the car and its driver use little signs of vehicle behavior such as “indicative” braking or hand gestures that help all parties in a decision-making situation (Moore et al., 2019), for example, to cross the street in front of a car. With the disappearance of a driver in a fully automated vehicle, signs by the driver no longer exist. At the same time, there is a clear understanding of the pedestrians that automated vehicles are highly regulated and have safety measures in place in case a pedestrian crosses their way (Millard-Ball, 2018; Holländer et al., 2019). Therefore, the question is, in which situations pedestrians would indeed exploit the latter when crossing a street by relying on the safety features of automated vehicles (AVs) and by enforcing the vehicle(s) to stop and to claim the right to cross the street. Hence, understanding how individuals will interact with AVs in urban traffic is still a key challenge on the path to autonomous driving (Tabone et al., 2021) before AVs can independently navigate our streets.

As an additional constraint, social challenges play a key part before they can travel the streets without continuous interference. Various news articles report incidents where vulnerable road users disturb AVs, ranging from simple negative gestures to pointing a gun (Condliffe, 2016; Randazzo, 2018; Brown, 2019; Keck, 2019). Those instances even led some companies to conduct their trials with unmarked AVs to prevent potential bullying by other road users (Connor, 2016). As these instances paint a rather grim picture of human-AV interaction, traffic interaction is not a one-shot game and does not occur in a social vacuum. Instead, it is embedded in a set of formal and informal (social) norms (Björklund and Å berg, 2005). Exploiting an AV, for example, by jaywalking in front of it, is, therefore, a specific form of human behavior often referred to as deviant behavior. Deviance refers to acts that break the social rules of those kinds of behaviors that are deemed acceptable by society. Deviant (rule-breaking) behavior is frequently sanctioned by society through social control (Brauer and Chaurand, 2010), a set of sanctioning and reward mechanisms that incentivize individuals to conform to societal expectations. These range from formal forms of sanctioning (e.g., laws and punishment) to social feedback in the form of, for example, positive reinforcement, shame, or ridicule. Social control could thus potentially moderate the rational incentive to exploit AVs. However, the moderating effect of social norms (*via* social control) on deviant behavior has received limited attention in the literature so far.

This study investigates how different forms of social control moderate pedestrians’ decision to jaywalk in front of AVs and human-driven vehicles (HDVs). Utilizing jaywalking behavior of pedestrians to study deviant behavior in the context of AVs has several benefits: 1) pedestrians benefit the most from a conflict-avoidant AV, drastically reducing their vulnerability in accident-prone situations, thereby increasing their utility to exploit them; 2) deviant behavior of pedestrians is commonplace in urban traffic situations, making it the most probable cause of interference for AVs; and 3) compared to other road users, the behavioral movement of pedestrians is significantly less predefined by the physical traffic environment, offering more frequent opportunities to act in line with self-interest.

### 1.1 Background

Road traffic is highly regulated in unclear traffic situations; drivers and VRUs use several forms of implicit and explicit communication ranging from deceleration up to hand gestures to let someone pass (Dey and Terken, 2017; Moore et al., 2019). Implicit or vehicle-centric (Dey et al., 2020a) communication cues can be summarized by vehicle movement patterns such as acceleration, deceleration, and vehicle distance (Varhelyi, 1998; Te Velde et al., 2005; Schmidt and Faerber, 2009; Risto et al., 2017). Explicit or driver-centric communication cues are managed *via* eye contact (Guéguen et al., 2015; Ren et al., 2016; Schneemann and Gohl, 2016; Nathanael et al., 2018) and gestures (Guéguen et al., 2015; Färber, 2016; Sucha et al., 2017) of the traffic participants.

With a disappearing driver in the automated vehicle, in unclear communication situations, the pedestrian would only have to rely on the vehicle-centric signals of the driverless vehicle alone. Research on pedestrian–AV interaction largely addressed this issue by exploring External Human–Machine Interfaces (eHMIs), which could assist communication between drivers and other traffic participants and could increase the acceptance of AVs (Carlsson and Nilsson, 2016; Chang et al., 2017; Dey et al., 2020b; Colley et al., 2022). Moreover, some other studies explored trust and overtrust of VRUs in AVs (Holländer et al., 2019; Jayaraman et al., 2019; Faas et al., 2020a; Holthausen et al., 2020).

Alongside acceptance and trust, one of the favorable measures for understanding interaction dynamics between AVs and pedestrians is the crossing decisions of participants. Faas et al. (2020b) emphasized the realistic walking behavior in related crossing paradigms rather than using a button or a safety slider for a better matching experience to realism. As a feasible solution, virtual reality (VR) has been widely used in pedestrian–AV interaction research because it allows for reproducible and controllable environments in immersive settings (De Clercq et al., 2019; Holländer et al., 2019; Jayaraman et al., 2019; Löcken et al., 2019; Mahadevan et al., 2019; Kalatian and Farooq, 2021). VR has also been effectively used in experimental paradigms where time pressure was tested in crossing tasks (Morrongiello et al., 2015; Schneider et al., 2019). Moreover, Bhagavathula et al. (2018) reported that pedestrian behavior was similar in VR compared to reality in terms of perceived safety and risk.

In order to reveal pedestrian crossing decisions in detail, Kalatian and Farooq (2021) conducted a large (*N* = 180) VR study. Their deep learning model emphasized the effect of AVs alongside street width, traffic density, and limited sight on elongated waiting times of pedestrians before crossing. In the VR cave study of Dommès et al. (2021), the authors tested the crossing behavior of pedestrians in front of conflict-avoidant AVs and conventional vehicles in a mixed traffic environment. They reported that participants were more hesitant to cross in front of AVs in some conditions. However, they also argued that participants mainly relied on locomotion cues of vehicles independent of their automation status. Jayaraman et al. (2019) conducted a gamified virtual reality study to investigate pedestrian trust in AVs in situations where AVs’ locomotion cues signalized aggressive, normal, and defensive behavior. Moreover, they controlled the traffic environment by testing pedestrian trust in unsignalized and signalized crossings with a traffic light. Their results indicated an increase in trust when AVs exhibited defensive behavior and when pedestrians were on signalized crossings. The work of Jayaraman et al. explored the aspects that can establish more pedestrian trust in AVs to encourage pedestrians to cross in front of AVs without hesitance. However, the long-term effects of trustworthy and defensive AV behaviors on individuals’ interaction with them are yet to be explored (Dommès et al., 2021).

Undeniably, human trust and the safety of AVs are essential before AVs are released on the streets. Nonetheless, some studies highlight the possible drawback of the conflict-avoidant behavior of AVs in their interaction with humans (Camara et al., 2018; Fox et al., 2018; Camara et al., 2020; Dommès et al., 2021). For instance, Moore et al. (2020) reported that human road users disturb driverless cars in a Wizard-of-Oz study with obstructive behavior types, ranging from playful curiosity to aggression to purposely stepping in front of them, which was also observed by Madigan et al. (2019). Similar behavioral patterns were also observed toward service robots by Salvini et al. (2010). Drawing on game theory, Fox et al. (2018) and Millard-Ball (2018) argued that if AVs are programmed with a zero-probability for collision, situations as these were to be expected: the shared argument is that a collision-avoidant AV will reduce other traffic participants’ risk of a crash or injury when interacting with them, thereby increasing the rational utility to exploit their passive stance for individual benefit, hence leading to a “freezing robot problem” in the mixed traffic of the future (Trautman and Krause, 2010). As a countermeasure, Camara and Fox (2020) introduced a pedestrian–AV interaction model where they suggested replacing conflict-avoidant AVs with a milder space invading AVs without introducing severe crash risks, inspired by findings regarding social factors in traffic among individuals.

One overlooked factor in AV–VRU research is social norms and social factors (Colley et al., 2019), alongside scalability problems (Colley et al., 2020; Dey et al., 2021). Pedestrians were more likely to cross the road if other pedestrians around them had started crossing (Faria et al., 2010). In a very recent study, Colley et al. (2022) tested the effects of pedestrian group behavior and a single pedestrian behavior on their participants’ crossing decisions in front of AVs, and they found similar results to Faria et al. (2010). However, there is still a large gap in exploring the social norms in AV–pedestrian research and carrying one-to-one interaction paradigms a step further.

### 1.2 Own Approach

In our study, we build on rational-choice theory, which assumes that individuals use their self-interests to make choices and model deviance as a function of an individuals’ cost-benefit calculation (Becker, 1968). In this context, deviant behavior occurs if the anticipated net gains from the specific action outweigh the anticipated losses associated with that action. This means exploiting the conflict-avoidant nature of AVs might only serve the self-interest of individuals, as it outweighs the costs of breaking social rules. Specifically, we focus on three different types of social control: 1) the “broken-window thesis” of a negative bystander effect, which should incentivize deviant behavior, 2) social conformity, moderating deviant behavior by conforming with societal expectations when in the presence of others, and 3) formal norm enforcement and sanctioning by authority.

Methodologically, we designed a 2 × 2 × 4 full-factorial VR experiment (vehicle type, task urgency, and social control), where individuals were asked to deliver pizza in a simulated urban traffic environment. We carried out the analysis in two parts: first, we tested for the effect of the experimental treatments under unknown probabilities that the human-driven car would stop, and then, we gave participants the possibility to signal the driver to stop, which succeeded 50% of the time. This way, we could likewise test whether the treatment effects depend on the lower crash risk when confronted with AV or whether potential effects might be caused by the autonomous nature of AV. Conducting this experiment in VR not only enabled us to obtain a closer approximation of the natural behavior of participants in a virtual environment (Deb et al., 2017a) but also offered time- and cost-effective testing setups where traffic situations could be built securely and flexibly. In our study, participants were faced with the choice to cross a busy road by jaywalking through a gap in traffic or wait until the traffic flow allowed for a safe and norm-compliant crossing. The first vehicle at the end of this gap was randomized to be either human-driven or an AV. Different social control conditions varied randomly by the presence of different road users with different characteristics and behaviors presented. Moreover, we manipulated the task urgency for the individual task as a third factor to test whether the moderating effect of social norms depends on the individual payoff for deviant behavior. Individuals were incentivized to cross the street by a small monetary reward.

The experiment employed a within-subjects design (*n* = 36), where every participant received all experimental treatment conditions. As repetitive crossing scenarios might potentially decrease motivation and increase task fatigue for participants Schneider et al. (2019), we employed gamification, a technique where participants are incentivized with various game elements such as badges or scores (Sailer et al., 2017).

Our research questions are formulated as follows:

• Are there differences between the crossing behavior of individuals when they encounter automated or conventional vehicles right after a traffic gap?
• Do positive, negative, and legal representations of social control cues affect the crossing behavior of individuals?
• Do different levels of task urgency-related time pressure affect the crossing behavior of individuals?

### 1.3 Contribution to the Field

Our study is timely concerned with newly emerging considerations in pedestrian–AV research. Firstly, we introduced a mixed traffic environment where both AVs and HDVs existed in the experimental scene. Secondly, we went out of the widely studied one-to-one interaction paradigms between AVs and pedestrians and contributed to limited scalability research in this area. Third, we explored potential social control mechanisms that can reduce or enhance the deviant behavior of pedestrians from three different dimensions: legal, positive, and negative norm cues. To our knowledge, such social control mechanisms were not a major focus in existing research, except for a negative example of a crossing pedestrian or idle pedestrian groups. Moreover, we tested legal norm cues under a study where the legal sanctioning was ambiguous, as opposed to studies that utilized definitive traffic lights or traffic signs. Forth, we further tested the effect of vehicle type and social control on deviant behavior when controlling for the risk of accidents for conventional vehicles. This allowed us to test whether significant differences between human-driven and autonomous vehicles existed, resulting from the autonomous nature of AVs and not their conflict-avoidant stance. Last but not least, our research contributes to the small sample of gamification literature in pedestrian–AV interactions, which supports a better-blinded method for repetitive within-subject designs.

## 2 Theoretical Framework

### 2.1 Exploiting Automated Vehicles as a Rational Decision

Recent studies on AV–pedestrian interaction draw on the game theory to argue that AV’s inability to adapt their behavior from a passive, conflict-avoiding stance would make incentive pedestrians step in front of them (Fox et al., 2018; Rahmati and Talebpour, 2018). Testing a sequential game of chicken, Fox et al. (2018), for example, suggested that assuming a zero-probability of collision between an AV and an HDV, based on the assumed conflict-avoidant programming, the expected cost of collision for the human driver likewise is nearly zero, which would result in the rational incentive to abuse AV for human drivers. Applying this model to the AV–pedestrian interaction and assuming the payoff structure to consist of the trade-off between time-savings and risk of personal injury while keeping the probability of crash at 0, we would receive the same result, even if the expected cost of a crash would be significantly higher for the pedestrian. Formally, this can be expressed by the expected utility theorem, which assumes that an individuals’ rational decision, given a set of possible alternative choices, is a function of the expected utility of the different choice options based on the probability distribution of the decisions’ outcomes. The decision to abuse an AV thus occurs if the expected utility of this choice is larger than or equal to the expected utility of alternative actions:

$$ExpectedUtility_{\mathrm{humanabuses}}=Utility_{\mathrm{abuse}}\;*\;Probability_{AVstops}>ExpectedUtility_{\mathrm{alternativeactions}}.$$

To illustrate this, we use the following hypothetical payoff matrix for the interaction between an AV and a pedestrian. We assume that, for each player, the utility to yield possesses a utility of −1 (lost time), whereas walking/driving possesses the utility of 1 (gained time). When both players choose to walk/drive, the result is a crash, which is significantly more costly to both players than the other choice outcomes.

We can then calculate the expected utility for a pedestrian to either walk or wait:

$$EU_{\mathrm{Walk}}=U_{Walk/AVyield}\;*\;p_{\mathrm{AV yield}}+U_{Walk/AVdrive}\;*\;\left(1-p_{AVdrive}\right)\;EU_{\mathrm{Wait}}=U_{Wait/AVyield}\;*\;p_{\mathrm{AV yield}}+U_{Wait/AVdrive}\;*\;(1-p_{AVdrive}).$$

Because *U*_*Wait*/*AVyield*_ = *U*_*Wait*/*AVdrive*_, which holds true for all possible payoffs as the cost to wait is independent of the choice of the vehicle:

$$EU_{\mathrm{Wait}}=U_{Wait/AVyield}=U_{Wait/AVdrive}=\text{−}1,$$

Given that *U*_*Wait*/*AVyield*_ = *U*_*Wait*/*AVdrive*_, the decision to cross then depends on the probability that the car will yield, which is a function of the utilities for the car yielding or driving when the pedestrian crosses. In this example,

$$EU_{\mathrm{Walk}}>EU_{\mathrm{Wait}}\mathrm{if}EU_{\mathrm{Walk}}>U_{Wait/AVyield}=U_{Wait/AVdrive}=\text{−}1,$$

which is true if *p*_*AVyield*_ > 99, 8%.

Given this minimalist payoff structure, the introduction of conflict-avoidant AVs would create a rational incentive for bullying AVs, as highlighted in previous studies (e.g., Fox et al., 2018; Millard-Ball, 2018). However, the utilities of traffic interaction in real life do not solely consist of the trade-off between time savings and risk of personal injury, which makes this model too narrow to reflect real-life behavior. For instance, traffic interaction (in most instances) is regulated by formal and informal rules.

### 2.2 The Cost of Norm Violation

Formally, traffic is regulated by traffic code, and to step in front of an AV would, in many instances, be considered a traffic violation, subject to fines and penalties. Similarly, even the AV/HDV interaction at an unmarked intersection used in the previous example would, in most jurisdictions, fall under the “priority to the right” rule. Informally, traffic is further regulated by social norms (including compliance with formal norms). Social norms generate a sense of predictability under uncertainty. In other words, social norms can be understood as equilibria of strategies to solve repetitive games, reducing the cost of uncertainty by believing that others will act in accordance with the norm. Frequent norm violations thus carry the risk of norm erosion, meaning that an established norm ceases to exist if individuals too frequently deviate from the said norm. The resulting norm erosion, in return, increases interaction costs by creating uncertainty with regard to the behavioral choices of other individuals in future interactions, which is not limited to the individual committing the norm violation but to society. Drawing on the previous example, if HDVs frequently violate the “priority to the right-” rule in the context of AVs, future interactions at unmarked intersections would be more time-consuming, as they would require individual negotiation between traffic participants because trust in norm compliance would be low, as the norm of “priority to the right” eroded.

Abusing or bullying a self-driving car, here in the form of jaywalking in front of it, is thus a form of human behavior commonly referred to as deviant behavior. Deviance describes actions or types of behavior that violate formal (i.e., laws and traffic code) or informal (i.e., social norms) rules (Goode and Ben-Yehuda, 2010). In other words, deviance refers to behavior that goes against what is deemed acceptable by society. Building on a rational-choice approach to deviance (Becker, 1968), we understand the associated norm violation as a function of an individual’s cost-benefit calculation and, following the expected utility theorem, expect deviant behavior to occur if the anticipated net gain from breaking the (formal or informal) rules outweighs the anticipated net gain from alternative actions. To be more specific, we build on the argument by Keuschnigg and Wolbring (2015) that a rule is rationally anticipated to be broken if the expected benefit of breaking this rule minus the cost of punishment (multiplied by the probability the rule-breaking will be sanctioned), is larger than the expected utility of alternative actions. The cost of norm violation then results from the incentive of other individuals to sanction norm violations (to prevent norm-erosion) and the cost of punishment, a mechanism often referred to as social control. While from the other perspective, norm compliance might also positively increase the utility of alternative actions (e.g., by intrinsic rewards). Adding the effect of social control to the utility function of jaywalking behavior, a person would then jaywalk if *EU*_*J*_ > *EU* and, thus, if

$$\left[U_{JS}*p_{S}+U_{JD}*\left(1-p_{S}\right)\right]U_{\mathrm{punishment}}*p_{\mathrm{sanctioning}}>\left[U_{WS}*p_{S}+U_{WD}*\left(1-p_{S}\right)\right]+U_{\mathrm{reward}}*p_{\mathrm{reward}}.$$

Note: *J* = jaywalking, *S* = vehicle stops, *W* = pedestrian waits, *D* = vehicle drives.

The decision to jaywalk would thus be influenced by three different components:

1) The individual gross utilities for the different choice options.
2) The probabilities for the individual choice outcomes to occur.
3) The cost of punishment and the probability of sanctioning.

Given that the moderating effect of norm compliance influences the net gains of the behavioral choice, all else being equal, its effect should be stronger in situations where the net gains are lower; that is, the expected utilities between the different choice options are more similar, compared to a more limited effect when the utility trade-offs between the choice options are higher. Hence, we would expect that an increase in utility for the deviant choice of jaywalking would increase the expected utility to jaywalk and therefore increase deviant behavior. We, therefore, expect the following:

H1 All else being equal, a higher utility payoff for the deviant behavioral choice will increase deviant behavior.

Because the expected utility of the deviant behavioral choice is dependent on the probability of occurrence of the different choice outcomes, we likewise expect that passively programmed AVs should increase deviant behavior, given that the probability the car will yield is programmed to be 100%.

H2 All else being equal, individuals will jaywalk more frequently when interacting with an AV.

The second hypothesis already implies that we do not expect social control (the cost of norm violation) to fundamentally alter the utility differences between interactions with AV and HDV, that is, social control to formally interact with vehicle type. This would be the case if the effect of social control would be substantially different for the individual vehicle types or specific social norms would exist that only apply to a specific type of vehicle. However, we are not aware of empirical evidence demonstrating that the cost of norm compliance significantly differs between HDV and AV or specific social norms that only apply to one type of vehicle. On the contrary, our main argument in this study is that social control applies to both HDV and AV and reduces the overall occurrence of deviant behavior, disregarding the vehicle type. In order to understand the extent of this moderating effect, it is important to differentiate between different forms of social control.

### 2.3 Social Control as a Moderator of Deviant Behavior

The influence of others on deviant behavior was formalized by Hirschi (1969) in the theory of social control. Hirschi viewed social sanctioning, which he explicitly differentiated from formal sanctioning, as an even higher deterrent of deviant behavior than formal rules (Hirschi and Gottfredson, 1994). Norm compliance, in return, results from individuals’ motivation to conform to social norms. More generally, social control refers to the rewards and sanctions that result from conforming to or deviating from social norms (formal or informal) (Ross, 1896). In line with this theory, research on red-light violations of pedestrians (Rosenbloom, 2009; Fraboni et al., 2018; Raoniar and Maurya, 2022) revealed that individuals cross with a higher frequency if they are alone, compared to situations where multiple individuals are waiting for the green light. Recent evidence suggests that this effect is further moderated by social proximity; it increases when individuals are surrounded by people they feel closer to or who belong to their social group.

H3a (Norm conformity) The presence of other pedestrians will decrease deviant behavior.

However, the presence of others can also have the opposite effect on deviant behavior. The observation of deviant behavior by other individuals incentivizes norm violations (Keizer et al., 2008). Formally, other individuals violating norms might serve as a cue that norms are not enforced in this area, or norm erosion occurs, which decreases the marginal cost for non-compliance. This effect exists even if the behavior of others has not been observed directly. However, the inference of low levels of norm compliance is made by social cues, such as littering, graffiti, or broken windows (“broken-windows thesis”) (Wilson and Kelling, 1982).

H3b (Negative bystander/broken windows) Cues signaling norm violations by others will increase deviant behavior.

Hirschi argues that social sanctioning serves as a higher deterrent to deviant behavior than formal norms, so evidence on traffic violations suggests that cues signaling the enforcement of formal norms have a strong negative effect on deviant behavior. Given the moderating effect of social proximity on norm-compliance, this might be explained by the larger social distance between individuals on public roads, which limits the effect of social sanctioning (e.g., a nasty look by a bystander is less costly than reproach by family members). In contrast, cues of formal norm enforcement and sanctioning make the cost of norm violation more salient for individuals.

H3c (Formal norm enforcement) Cues signaling sanctioning of formal norms will have a negative effect on deviant behavior.

## 3 Materials and Methods

### 3.1 Virtual Reality Environment

**FIGURE 2:**
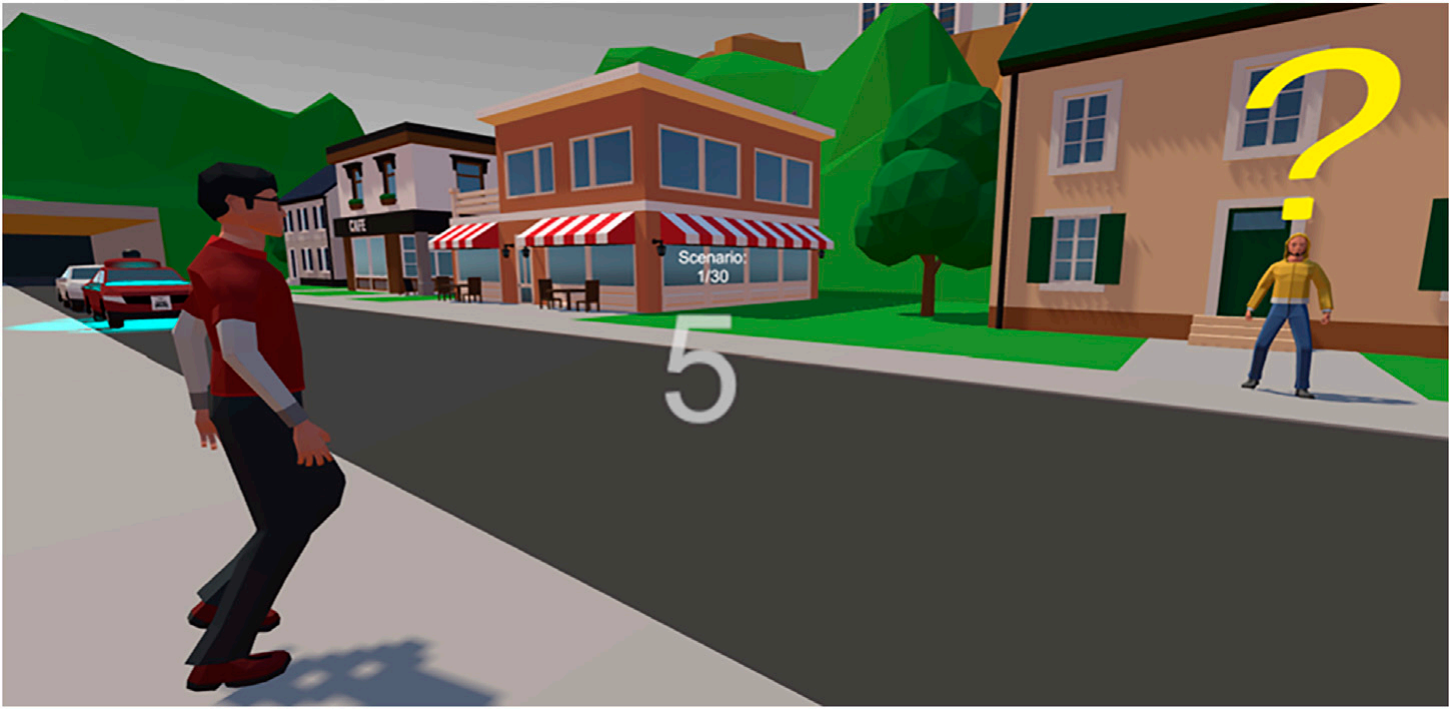
Virtual reality “street-crossing game” (participant perspective). Note: the participant is given the task of delivering pizza to a non-player character across the road. On the left side of the participant, a non-player character attempts to cross the road. A yielding AV can be spotted with blue deceleration light cues. The timer in the middle indicates 5 s left to earn the extra tip from pizza delivery.

**FIGURE 3:**
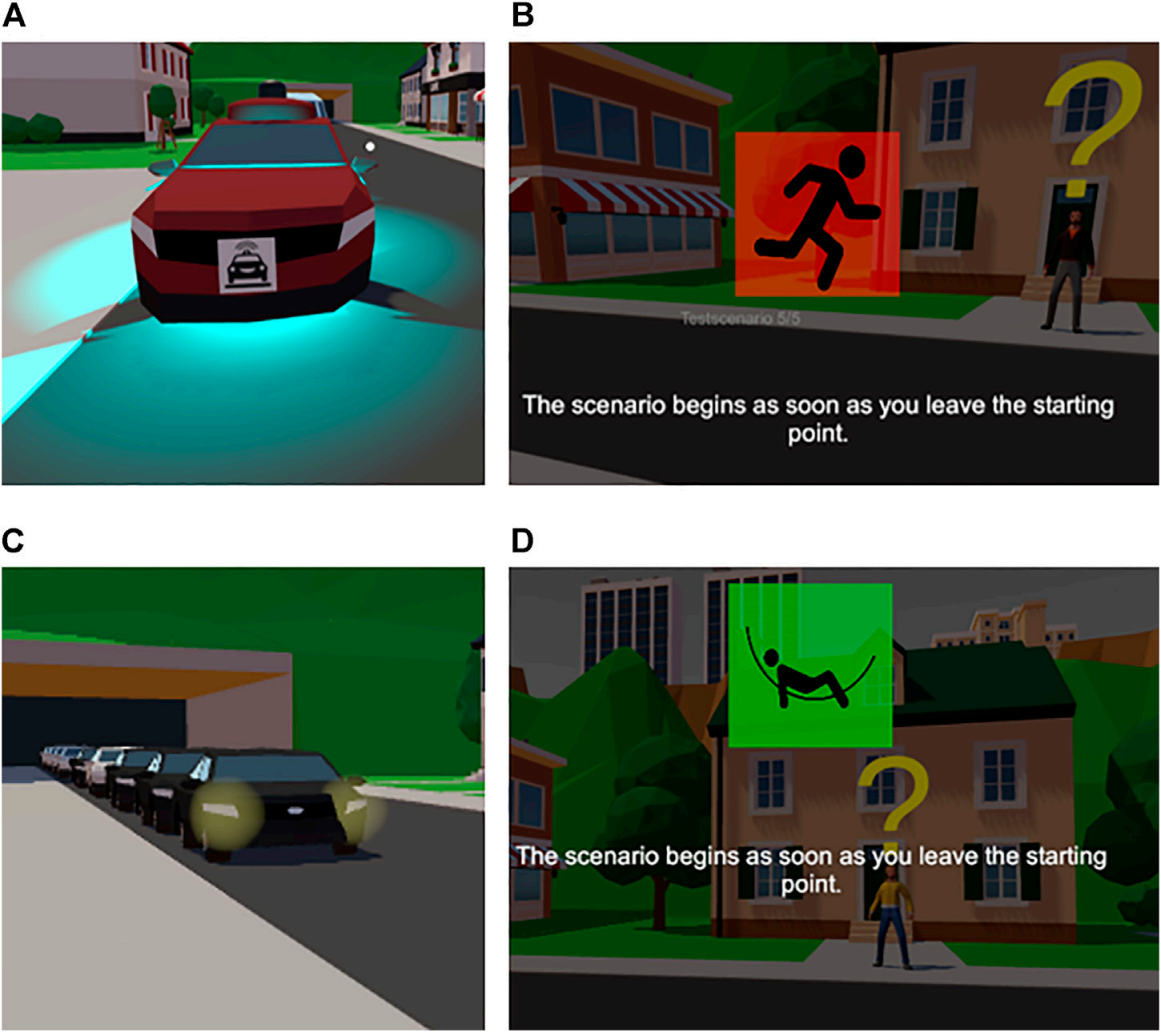
Vehicle type and task urgency factor levels in the experiment. **(A)** Decelerating AV casts blue light cues. **(B)** Urgent task indicator with a running man on a red background. **(C)** Decelerating HDV flashes headlights. **(D)** Non-urgent task indicator with a resting man on a green background.

**FIGURE 4:**
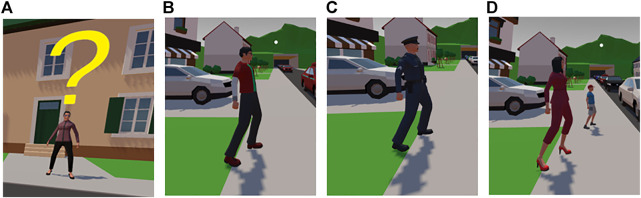
Non-player characters. **(A)** Target customer waiting for pizza delivery. **(B)** A walking person who crosses the road represents negative social control. **(C)** Police officer representing legal control. **(D)** Mother and child representing positive social control of abiding by the rules.

To conduct this experiment, we designed a virtual street environment in Unity 3D (version 2020.3.0f1). VR served as a flexible and safe test bed for running our study (see Figure 1B). The environment was limited by tunnels on both sides of the road and surrounded by hills. Urban buildings were placed on both sides of the street. Because we used game elements in our experiment, we did not focus on making the virtual environment realistic and utilized low polygon mesh elements (see Figures 2–4). The placement of traffic signs, pedestrian crossings, and traffic lights intentionally abstained so that participants could only use the information of vehicle movements and communication cues on their crossing decisions. The size of the street, including pavement, was 12 m. Participants emerged a few steps away from the sidewalk while traffic was flowing on the road. The unidirectional traffic coming from the left side of the participant consisted of fully automated and conventional vehicles. Vehicles had a 50 km/h start speed and exponential deceleration behavior with starting value of 1.98 km/h. Vehicles stopped at a sufficient distance to provide a traffic gap for participants to cross. Virtual human characters emerged on the left side of the participant when they accompanied the scene. This allowed both oncoming vehicles and virtual road users to be in the participants’ field of view (see Figure 2).

The task of the participants was to score points by delivering pizza to a virtual character waiting on the opposite side of the road (see Figure 4A). If participants failed to deliver pizza for reasons such as getting caught by the police, they did not receive any points. Otherwise, they either received 1 base point for delivering the pizza or 2 points for delivering the pizza within the bonus timer. The traffic pattern consisted of two waves of vehicles passing the scene from left to right. Between the first and second waves of vehicles, a gap of around 3 s opened up. Participants were then faced with the choice to either jaywalk in this situation or wait until the second wave of cars passed.

### 3.2 Experimental Design

Our experiment consisted of three factors (vehicle type, task urgency, and social control) with different factor levels, resulting in a 2 × 2 × 4 full-factorial design, where all experimental conditions varied randomly within subjects. This design provided control for individual differences; it allowed us to examine the effect of multiple independent variables and their interactions at a time, and it was more efficient because smaller sample sizes could be sufficient for statistical power. The experimental treatments consisted of the combinations of the different factorial levels that we operationalized by manipulating specific elements of the individual scenes.

#### 3.2.1 Vehicle Type

To understand the differences in crossing behavior between self-driving and conventional vehicles, we manipulated the first vehicle of the second wave of cars to be either an AV or an HDV. To increase the realism of the situation and understand whether the crossing decisions are dependent on a lack of communication between the pedestrian and vehicle, we operationalized the HDV condition in two ways: equal amounts of conditions with a successful communication between the driver and the pedestrian when participants tried to negotiate for the right of way and conditions with conventional vehicles that did not respond to negotiation request.

AVs always yielded to participants as soon as participants stepped onto the road, so we could simulate their defensive design principles. For sending feedback to participants, AVs switched on a light-blue light when they started decelerating (Werner, 2018) (see Figure 3A). Conventional vehicles stopped for the participant if the participant performed a hand gesture coupled with a button press and the vehicle was a part of the successful communication subset. This gesture represented the explicit communication between the vulnerable road users and drivers. For sending deceleration feedback, HDVs flashed their headlights to participants (see Figure 3C). In the failed-communication subset, HDVs neither stopped nor indicated other forms of cues to participants. Participants were unaware of the types of conventional vehicles, and they were only informed that human drivers may or may not respond to them.

#### 3.2.2 Social Control

To understand the effect of different forms of social control on crossing behavior, next to the baseline condition of no social control, we tested for the effect of social conformity, cues indicating formal norm enforcement, and the effect of a negative bystander. To represent different social controls, we placed virtual human characters on the left side of the participant (see Figures 4B–D). For representing a positive norm of social conformity, a mother and a child waited before crossing until all vehicles passed. A mother and her child were chosen for this condition, as the social norm of rule compliance should be stronger when acting as a possible role model for the child. The negative bystander/broken-windows condition was operationalized by a walking person who stopped the oncoming vehicle wave after the small traffic gap was used. Formal norm enforcement and possible sanctioning were operationalized by the presence of a police officer. Participants were informed that police may or may not see them. If police saw them attempting to cross by obstructing the traffic flow, participants were stopped; hence, they received 0 points from that trial. This game mechanism represented a subtle cost of legal punishment. Because crossing the road in our scenario was not illegal, we avoided any direct punishment implications. In order to reduce the bias of police behavior, we sat up equal amounts of catching and non-catching police conditions in the design.

#### 3.2.3 Task Urgency

To understand the effect of different payoffs on jaywalking behavior, we tested for the effect of different task urgency and different payouts for jaywalking. This factor consisted of two levels: urgent and non-urgent. Urgency levels were cued with symbols before each trial started (see Figures 3B,D). In the scenario, participants received 1 base point for successful pizza delivery. However, they could double their earnings when completing the task in the set time frame. Therefore, scenarios were presented with a timer indicating the remaining time for earning a bonus point (see Figure 2). In non-urgent trials, the bonus timer started counting back from 23 s, which was enough for waiting until all vehicles passed, and it was safe to cross. In this condition, individuals received 2 points (base + bonus), disregarding their crossing decision. In urgent trials, the bonus timer started counting back from 13 s, meaning that participants had to jaywalk in front of a vehicle to complete the task with 2 points.

### 3.3 Collected Measures

As dependent variables, we collected both the crossing decision of individuals and the associated crossing onsets. Crossing onsets captured the time passed from the moment a trial started until a participant stepped on the road (in seconds). The crossing decision was observed by the researchers and was cross-checked with the collected crossing onsets, which were filtered by a series of criteria. First, participants crossed if the crossing onsets were smaller than the time needed for the last car of the second wave of cars to pass an invisible line. Second, if the last car could not reach the invisible line before the participant, either the participant successfully reached the other side or because a crash occurred (See Supplementary Material).

Because we implemented a second choice task for HDVs, to test for the effect of vehicle type and social control under equal risk of collision between human-driven and automated vehicles, we then split the dependent variable of crossing onsets into two. For the general differences, we only used those observations where the crossing decision was made within 1 s after the first wave of cars passed (7.75 s), which equals around 1 s before the second wave’s arrival. This point is likewise below the reaction time of the risk-controlled, yielding signaling its intention to stop. For those observations, we could logically assume that the crossing decisions for scenarios with an HDV were made, disregarding the behavior of the other vehicle and under unknown probabilities of a collision. To compare the crossing decision under equal risk for a crash, we used all observations where the participant crossed later than the initial time frame, crossed or did not cross when interacting with an AV, or elected to not cross when faced with an HDV where successful negotiation could have been possible (which was unknown to the participant, but signalized that no attempt to stop the car was made). As independent variables, we used the experimental treatment conditions and coded them into three factors (vehicle type, task urgency, and social control).

After finishing the VR experiment, participants filled out an online survey in LimeSurvey (version 3.27.26) Schmitz (2012) consisting of the IGroup Presence Questionnaire (IPQ) (Schubert et al., 2001), a demographics form (Deb et al., 2017b), the Pedestrian Receptivity Questionnaire for Fully Autonomous Vehicles (PRQF) (Deb et al., 2017b), the Pedestrian Behavior Questionnaire (PBQ—Short Version) (Deb et al., 2017b), and the Social Value Orientation (SVO) (Murphy et al., 2011) scale. Within the scope of this study, we have only used these measures to draw a clearer participant profile, and we did not evaluate them further in statistical analysis. Lastly, we presented five open questions regarding the effects of manipulated factors in the experiment (see Appendix).

### 3.4 Participants

Thirty-six participants (21 females, age: *M* = 25.22, ± *SD* = 5.15) were recruited *via* the online notice board of the university and printed “Pizza Delivery Game” advertisements on bus stops. Participants were informed they would be reimbursed with 8–10 euros, depending on the final game score. However, all participants eventually received a compensation of 10 euros for their participation, which was revealed at the end of the experiment. The Ethics Committee of the University of Oldenburg gave ethical approval for the experiment according to the Declaration of Helsinki.

**FIGURE 5:**
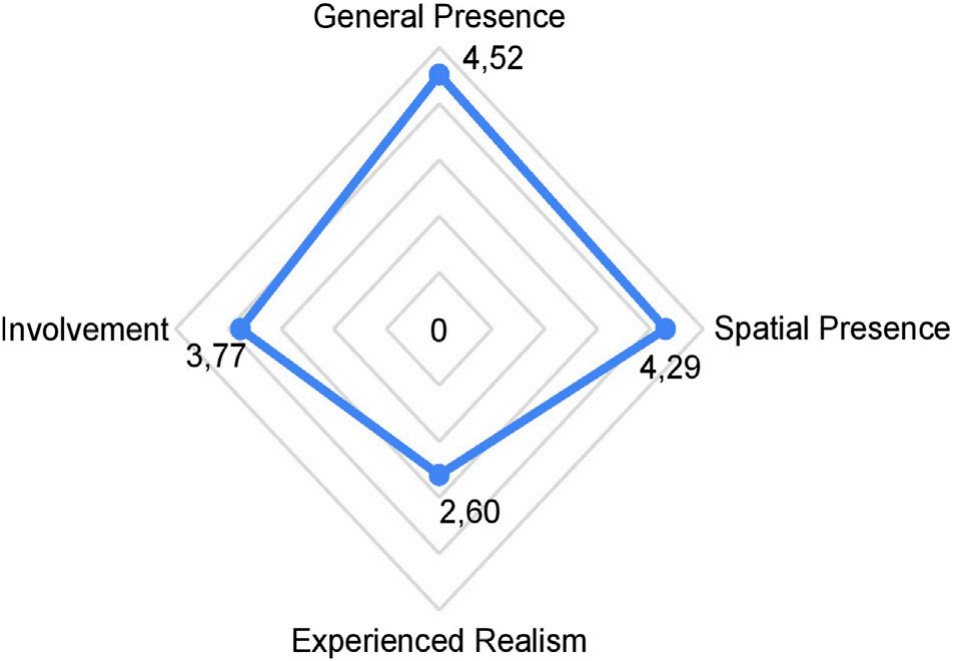
iGroup Presence Questionnaire Evaluation. Note: iGroup Presence Questionnaire Evaluation with means of the subscales involvement, experienced realism, spatial presence, and general presence.

Most participants reside in big cities with an overall population density of at least 193 people per square km. Most of them were high school graduates (*n* = 14) or graduate students (*n* = 10). Thirty-two participants would fall in the prosocial category on the Social Value Orientation angle (*M* = 32.69, ± *SD* = 8.77) (Murphy et al., 2011). Their (PRQF) (Deb et al., 2017b) grand scores had a mean more on the positive side of the scale (*M* = 66.63, ± *SD* = 10.88), indicative of greater receptivity for AVs. The average PBQ-Short Version (Deb et al., 2017b) grand score of the participants was 43.08, on the negative side of the scale, indicating safer pedestrian behavior (±*SD* = 6.80). Inspection of the IGroup Presence Questionnaire (IPQ) (Schubert et al., 2001) revealed high general presence (*M* = 4.52, ± SD = 1.20), high spatial presence (*M* = 4.29, ±SD = 0.97) and above-average involvement *M* = 3.77, ±SD = 1.12) in our VR experiment. However, experienced realism was rated on the negative side of the scale (*M* = 2.60, ±SD = 0.74) (see Figure 5).

### 3.5 Experimental Procedure

Participants were invited to a large meeting room. This provided enough space for walking a street-long distance of 12 m (see Figure 1B). First, participants gave their written consent and received specific information about the study and the associated task. Secondly, they were introduced to the Oculus Quest 2 VR headset and controllers (Facebook Technologies, LLC.). Then, they were instructed about the virtual guardian walls that indicate safe zones in the real environment. The virtual environment was re-positioned in a way that participants could walk straight to the virtual customer within the safe zone.

Before the experiment started, each participant conducted five test trials to familiarize themself with the environment, as in Jayaraman et al. (2019) and Kalatian and Farooq (2021). In the first trial, participants experienced crashing and dying, where they received the information about dying with a text on black background. They were also falsely informed that if they died in the experiment, the experiment would be over without earning the extra incentive. We gave this information to increase the cost of dying in the game. In the second trial, participants tried to stop the conventional cars by communicating with a gesture combined with a button press on the controller. The third trial showed them that conventional vehicles do not always consider their requests, and they keep on driving. In this trial, they also saw a very large traffic gap where the road was free of vehicles. They were reminded that this gap existed in each trial. The last two test trials were dedicated to police conditions where a policeman is either aware or unaware of the participant. In these last two trials, participants also practiced crossing in front of an AV. After making sure participants had no questions, 30 pseudo-randomized experimental trials began. Lastly, participants filled out online survey questions at the end. The virtual reality experiment took, on average, 30 min, in line with Kalatian and Farooq (2021) due to the increase in fatigue after 30 min, and the survey took 30–40 min to complete.

### 3.6 Analytical Approach

Before conducting the analysis, we ran a series of validity checks and excluded observations that were either implausible or instances where participants did not start crossing due to rare bugs. These include instances where respondents were free-falling from the environment or the trial time was elapsed. Unusual crossing onsets smaller than 1 s and bigger than 20 s (4/864) were ignored, resulting in a final sample size of *N* = 36 with 860 observations.

To understand the effect of the experimental treatments on the crossing behavior, we calculated a generalized linear mixed-effects model (GLMM) (Nelder and Wedderburn, 1972), including the experimental factors as fixed effects and treating within-subject variance as random effects. The crossing behavior of individuals served as a binomial dependent variable in the analysis, which we regressed on dummy variables for the experimental factors. We tested for both the main effects of the three experimental factors and interaction effects between vehicle type and both social control and task urgency. The statistical analysis was performed in RStudio (version 1.4.1106) (R Studio Team, 2020), using the glmer function of the Lme4 package (version 1.1-27.1) (Bates et al., 2015). The distribution of residuals in our models was cross-checked with the check_distribution function of the R performance package (version 0.8.0) (Lüdecke et al., 2021). Model fittings were tested *via* the base ANOVA function of R with Chi-squared tests and compare_performance function in the performance package. We also report the predicted marginal effects of each condition with crossing probabilities, which were calculated using the ggeffects package (version 1.1.1.1) (Lüdecke, 2018). They are reported in percentages after the multiplication of 100. Marginal effects indicate the average treatment effect of our experimental factors (or interaction of factors), holding the other factors constant in their proportions.

## 4 Results

In this part, we report the results of the experiment, both for the baseline experiment under unknown risk of a crash with an HDV (see Section 4.1) and a second analysis using a subset of risk-controlled crossing decisions, where participants were able to stop the HDV (see Section 4.2). Section 4.1 includes crossing attempts in front of HDVs where participants did not try to negotiate with the driver. Section 4.2 excludes these trials and demonstrates the results of participants when they negotiated with HDVs and when they tried to stop the vehicles by communicating with the drivers with a gesture. We have made this two-level analysis to observe the overall effect of vehicle types on our study and the pure effect of vehicle automation on crossing behavior when the risk of crashing is eliminated for HDVs.

For reporting the main effects, we elected to present the average marginal effect of the experimental factors, which is the effect of the factor levels of interest in reference to the baseline level, while holding the other factors constant at their proportions and the marginal means, which is the average crossing probability of participants when holding the other factors constant at their proportions. As the average marginal effect helps illustrate the causal effect in reference to the reference level, the marginal means illustrate the overall descriptive means for the different treatment conditions. We chose to report marginal effects because they are more intuitively understandable than odds ratios, reporting changes in or the overall means of crossing decisions for the different treatment conditions in percentages.

Overall, participants chose to cross deviantly in 62.1% of the trials, whereas in 37.9% of the cases, they decided to wait. The crossing decisions were most common when confronted with an AV, where they chose to cross in 71.4% of the trials, whereas when confronted with an HDV, only 57.4% elected to cross. When faced with an HDV, the crossing decision was equally distributed between observations where participants did not know about the probability that the car would stop (27.7%) and trials where participants successfully signaled the car to stop (29.7%).

### 4.1 General Crossing Predictions

**FIGURE 6:**
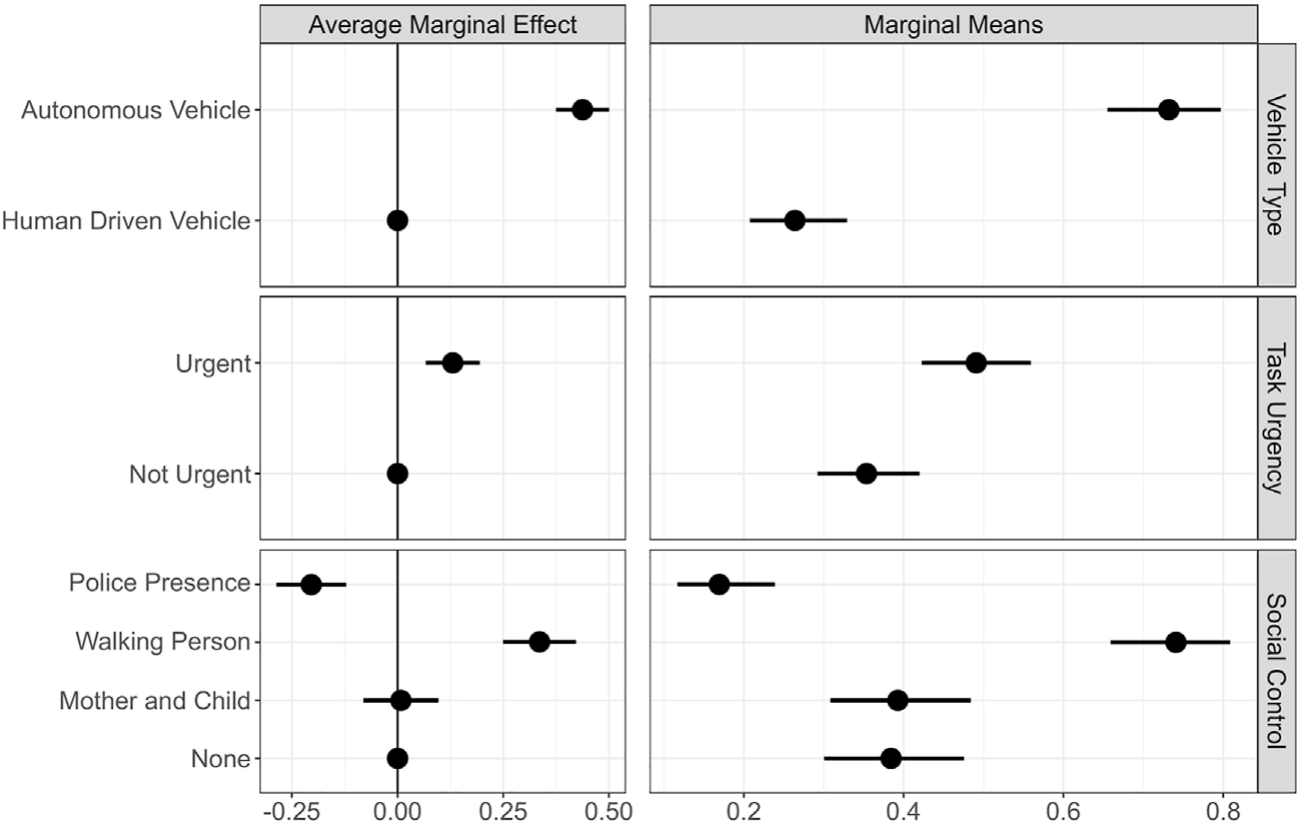
Average marginal effects and marginal means for general crossing. Note: left plot shows the average marginal effects (AME) of our three experimental factors in reference to their baseline factor levels. The vertical line represents the effect of the reference level. The right column reports the marginal means (MM) for the different factor levels on crossing probabilities, holding the other factors constant at their proportions. Points indicate AME/MM, horizontal lines the 95% CIs. Effects based on results of GLMM.

The results of the Generalized Linear Mixed Effects Model to model individuals’ general crossing decisions are provided in Table 1, and the predicted marginal effects and marginal means for the different treatments are illustrated in Figure 6. We used distinctive models to calculate the marginal effects. While models 1, 2, and 3 show the results for the main effects of vehicle type, task urgency, and social control, respectively, models 4 and 5 indicate the interaction between vehicle type × task urgency and vehicle type × social control.

**TABLE 1:** General results for the effect of vehicle type, task urgency, and social control on crossing decisions.

Predictors	M1 odds ratios	M2 odds ratios	M3 odds ratios	M4 odds ratios	M5 odds ratios
(Intercept)	0.36***	0.55***	0.62*	0.25***	0.23***
Autonomous vehicle	7.61***			7.94***	14.68***
Urgent		1.76***		2.01***	
Walking person			4.58***		6.85***
Police presence			0.33***		0.31**
Mother and child			1.04		1.06
Autonomous vehicle * urgent				1.04	
Autonomous vehicle * walking person					2,233,228.92
Autonomous vehicle * police presence					0.56
Autonomous vehicle * mother and child					1.02
Random effects					
*σ* ^2^	3.29	3.29	3.29	3.29	3.29
*τ*_00_	0.57	0.35	0.54	0.61	1.08
ICC	0.15	0.10	0.14	0.16	0.25
*N*	36	36	36	36	36
Observations	860	860	860	860	860
Marginal *r* ^2^/conditional *r* ^2^	0.192/0.312	0.022/0.117	0.187/0.301	0.220/0.342	0.865/0.898

***p < 0.001, **p < 0.01, *p < 0.05.

Note: results of generalized mixed-effect regression models. Odds ratios and random effects are reported for models 1–5. M1: vehicle type, M2: task urgency, M3: social control, M4: vehicle type × task urgency, M5: vehicle type × social control.

With all else being equal and keeping the effect of the other factors constant at their proportions, we find the presence of AV to significantly increase the crossing probability by 43% in comparison to HDV (*β* = 2.03, *z* (860) = 12.01, Pr (>|*z*|) < 0.001) (see Figure 6 top left). Overall, this meant for our participants that the average probability of crossing increased from 26% when interacting with an HDV to around 73% when interacting with an AV (see Figure 6 top right). Similarly, in reference to non-urgent scenarios, urgent scenarios significantly increased average probability of crossing by 13% (*β* = 0.56, *z* (860) = 3.95, Pr (>|*z*|) < 0.001) (see Figure 6 middle left). The average probability of crossing in urgent scenarios was 49%, whereas it was 35% in non-urgent scenarios (see Figure 6, middle right). Lastly, when contrasted to the baseline social control condition of being alone, the presence of a police significantly reduced the crossing probability by 20% (*β* = −1.11, *z* (860) = -4.77, Pr (>|*z*|) < 0.001); the presence of a walking person significantly increased crossing probability by 33% (*β* = 1.52, *z* (860) = 7.06, Pr (>|*z*|) < 0.001); and the bystanders mother and child did not change the probability of crossing (*β* = 0.03, *z* (860) = 0.17, Pr (>|*z*|) = 0.86) (see Figure 6 bottom left). Our participants’ crossing probability was predicted as 16% in the presence of police. Moreover, an increase of 74% was observed when accompanied by a walking person who attempted to cross the road. With mother and child condition, the crossing probability was at 39%. Finally, when the participants were alone in the scene, their crossing probability was 38% (see Figure 6, bottom right).

### 4.2 Risk Controlled Crossing Predictions

Since participants were unaware of the probability that an HDV would stop for the initial crossing decision, the strong effect of AV on the crossing decision might be caused by their passive programming and autonomous nature. To test whether the decision to cross is influenced by their autonomous nature and whether the effect of social control changes under equal risk distributions between AV and HDV, we conducted a second analysis, excluding those observations where the risk of a crash with an HDV was unknown.

**FIGURE 7:**
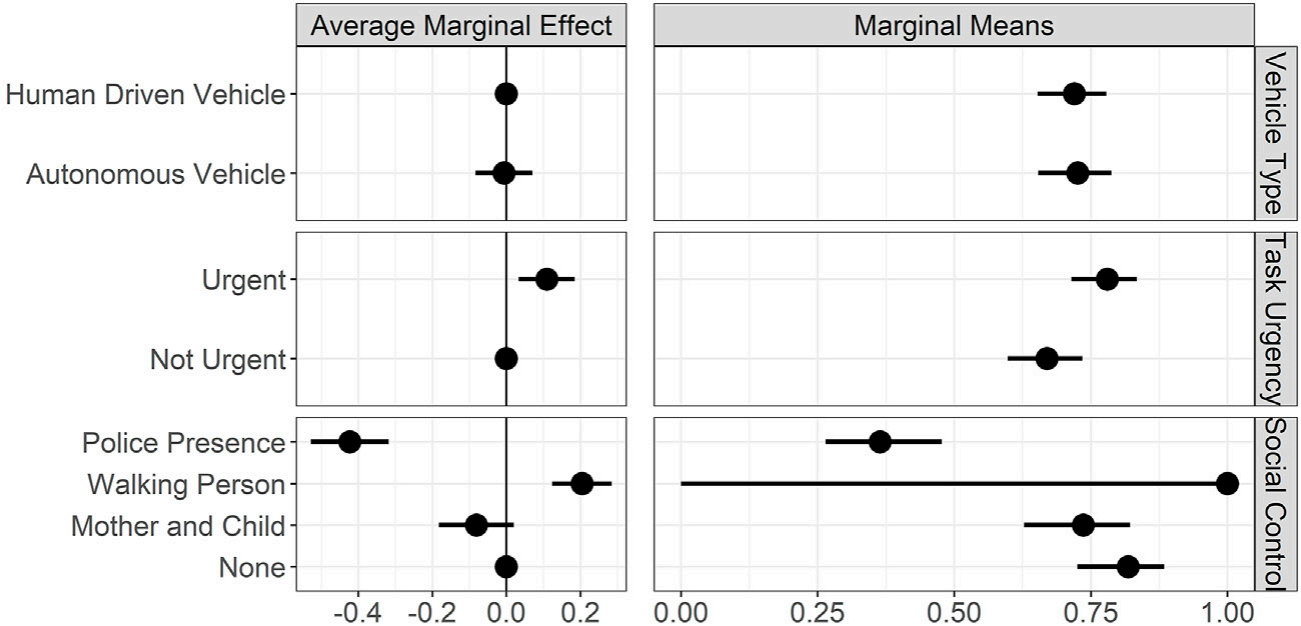
Average marginal effects and marginal means for risk-controlled crossings. Note: left plot shows the average marginal effects (AME) of our three experimental factors in reference to their baseline factor levels. The vertical line represents the effect of the reference level. The right column reports the marginal means (MM) for the different factor levels on crossing probabilities, holding the other factors constant at their proportions. Points indicate AME/MM, horizontal lines the 95% CIs. Effects based on results of GLMM.

The results of the Generalized Linear Mixed Effects Model to model individuals’ risk-controlled crossing decisions are provided in Table 2, and the average marginal effects and marginal means for the different treatments are illustrated in Figure 7. Similar to Table 1, models 1, 2, and 3 show the results for the main effects of vehicle type, task urgency, and social control. Models 4 and 5 indicate the interaction between vehicle type × task urgency and vehicle type × social control.

**TABLE 2:** Risk-controlled results for the effect of vehicle type, task urgency, and social control on crossing decisions.

Predictors	M1 odds ratios	M2 odds ratios	M3 odds ratios	M4 odds ratios	M5 odds ratios
(Intercept)	2.65***	2.03***	4.50***	2.25***	8.13***
Autonomous vehicle	0.97			0.82	0.38*
Urgent		1.75**		1.47	
Walking person			79,112,259.30		44,516,415.95
Police presence			0.13***		0.07***
Mother and child			0.62		0.28*
Autonomous vehicle * urgent				1.38	
Autonomous vehicle * walking person					2.64
Autonomous vehicle * police presence					2.57
Autonomous vehicle * mother and child					3.81*
Random effects					
*σ* ^2^	3.29	3.29	3.29	3.29	3.29
*τ*_00_	0.28	0.29	0.79	0.30	0.83
ICC	0.08	0.08	0.19	0.08	0.20
*N*	36	36	36	36	36
Observations	524	524	524	524	524
Marginal *r* ^2^/conditional *r* ^2^	0.000/0.079	0.021/0.102	0.944/0.955	0.023/0.105	0.944/0.955

***p < 0.001, **p < 0.01, *p < 0.05.

Note: odds ratios and random effects are reported for models 1–5. M1: vehicle type, M2: task urgency, M3: social control, M4: vehicle type × task urgency, M5: vehicle type × social control.

**Table:** 

		*Pedestrian*	
		*Wait*	*Walk*
*Autonomous Vehicle*	*Yield*	(−1, −1)	(−1, 1)
	*Drive*	(1, −1)	(−100, −1,000)

With all else being equal and holding the effect of other factors constant at their proportions, we see no effect of AV compared to HDV when we controlled for the risk (*β* = −0.03, *z* (524) = -0.15, Pr (>|*z*|) = 0.87) (see Figure 7, top left). When the crossing probability in front of AVs was 71%, the crossing probability in front of HDVs was 72% (see Figure 7, top right). The effect of urgency remained significant when crossings were controlled for the risk. Compared to non-urgent scenarios, urgent scenarios increased crossing probabilities by 10% (*β* = 0.55, *z* (524) = 2.78, Pr (>|*z*|) < 0.01) (see Figure 7, middle left). Their own effect on crossing probabilities was observed as 78% for urgent and 66% for non-urgent scenarios; Figure 7; middle right). Compared to baseline social control condition, as police presence significantly decreased crossing probabilities by 42% (*β* = −2.05, *z* (524) = −6.97, Pr (>|*z*|) < 0.001), the walking person increased it by 20%, which was not significant (*β* = 18.18, *z* (524) = 0.020, Pr (>|*z*|) = 0.98). Mother and child lead to a decrease in 8%, which remained insignificant (*β* = −0.47, *z* (524) = −1.56, Pr (>|*z*|) = 0.11) (see Figure 7, bottom left). The effect of social control levels on crossing probability, when kept constant at their proportions, was observed to be 36% for police presence, 100% for the walking person, 73% for mother and child, and, lastly, 81% when participants were alone in the scene (see Figure 7, bottom right).

### 4.3 Exploring Interactions

**FIGURE 8:**
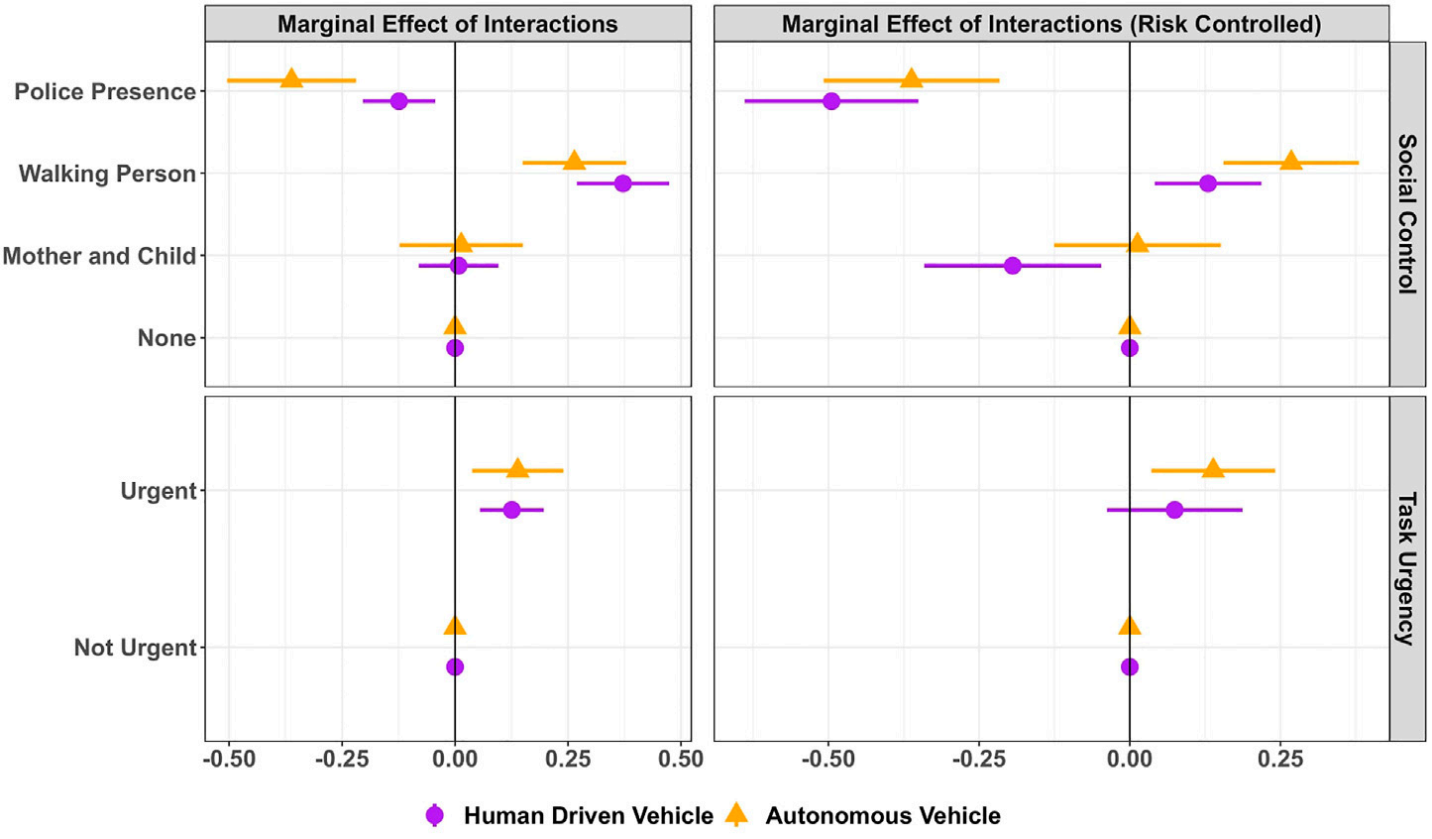
Effect of social control and task urgency conditioned by vehicle type. Note: the figure illustrates the average marginal effects on crossing probabilities of social control and task urgency, conditioned on vehicle type, both for the baseline crossing decision under uncertainty of HDV behavior (left side) and interactions where participants were faced with the equal risk of collision between AV and HDV (right side). Purple points represent HDV, and orange triangles represent AV. Horizontal lines show 95% CIs. Vertical lines represent the average crossing probability of the reference level.

Given the lack of empirical evidence on a potential interaction effect between social control and vehicle type, that is, whether social control might have a different effect on AV compared to HDV, we further explored potential interactions with GLMM models 4 and 5 for general crossings at Table 1 and risk-controlled crossing at Table 2. The average marginal effects for the interactions, AMEs of Social Control and Task Urgency conditioned on vehicle type, are illustrated in Figure 8.

When general crossings are considered, compared to being alone, the presence of police decreased crossing in front of AVs by 36% and HDVs by 12%. This interaction was not significant (*β* = −0.58, *z* (860) = −1.07, Pr (>|*z*|) = 0.28). Walking person increased crossing probability in front of AVs by 26% and HDVs by 37%. However, this interaction was also insignificant (*β* = 14.61, *z* (860) = 0.03, Pr (>|*z*|) = 0.97). Mother and child had an effect of increasing crossing probability in front of AVs by 0% and HDVs by 1%, which was an insignificant result (*β* = 0.02, *z* (860) = 0.04, Pr (>|*z*|) < 0.96) (see Figure 8, top left).

When we controlled for the risk and checked the interaction of vehicle type × social control, compared to being alone, police presence decreased the crossing probability in front of AVs by 36% and HDVs by 49%. However, this interaction was not significant (*β* = 0.94, *z* (524) = 1.54, Pr (>|*z*|) = 0.12). The walking person increased the crossing probability in front of AVs by 26% and HDVs by 12%. The interaction was not significant (*β* = 0.97, *z* (524) = 0.001, Pr (>|*z*|) = 0.99). The mother and child condition increased crossing probability in front of AVs by 1% and decreased the crossing probability in front of HDVs by 19% and this interaction was significant (*β* = 1.33, *z* (524) = 2.09, Pr (>|*z*|) < 0.05) (see Figure 8, top right).

The interaction of vehicle type by task urgency did not yield significant results in both general and risk-controlled results. Considering general crossings and compared to non-urgent situations, in urgent scenarios, participants’ crossing probability in front of AVs increased by 12% and in front of HDVs by 13% (*β* = 0.03, *z* (860) = 0.10, Pr (>|*z*|) = 0.91) (see Figure 8, bottom left). When controlled for risk for the same interactions, participants’ crossing probability in front of AVs increased by 13% and HDVs by 7% (*β* = 0.32, *z* (524) = 0.79, Pr (>|*z*|) = 0.42) (see Figure 8, bottom right).

## 5 Discussion and Conclusion

Will individuals bully or abuse AVs for individual gain? We had run a two-step analysis in the results section where we tested crossing decisions when the anticipated risk for AV was low and the anticipated risk for HDV was higher in the first step. This step mimicked the expected future mixed traffic environment with imbalanced costs of exploiting an HDV and an AV. Our results indicated a higher deviant behavior toward AVs when the risk distribution was not balanced. These results support the findings of Moore et al. (2020), in which they observed deviant behavior toward self-driving vehicles in their field observation. Moreover, our results are also corroborated by remarks from our respondents. When we asked whether different vehicle types influenced their crossing behavior, more than half of the answers indicated an existing effect. Participants stated that they crossed the street “without hesitation” in the presence of AVs, relying on the passive stance of AVs, and they were more willing to cross in front of AVs. One respondent explained in AV conditions that he crossed even without waiting for the blue deceleration signal of AVs. These results are in the direction of “Overtrust” toward AVs problem, as Holländer et al. (2019) argued. However, in the second step of the analysis, when we balanced the risk distribution by only including HDV trials where HDVs could yield if participants negotiated with them, our data could tell if there were remaining differences in crossing behavior stemming from the sole effect of automation attributes of vehicles. As we ran the analysis, we observed that the existing difference between crossing predictions among HDVs and AVs simultaneously disappeared when the crash risk of HDVs disappeared. These results emphasize the importance of risk avoidance in participants’ crossing decisions more than the automation status of vehicles, which is in line with the remarks of Dommès et al. (2021) that pedestrians rely mainly on vehicle dynamics and locomotion cues before taking a crossing decision. Therefore, we can only confirm H2 that when the collision risk is introduced in HDVs when AVs stay risk-free, deviant behavior toward AVs increases, as Millard-Ball (2018) anticipated with his game theory-derived remarks.

Kalatian and Farooq (2021) observed in their VR study derived models that pedestrians’ waiting time before crossing was longer in mixed traffic and only AV scenarios than in only HDV scenarios. Their study did not report trials where vehicles did not stop; hence, the risk distribution among vehicle type levels seemed equal. When we compare their results with our risk-controlled crossings, we fail to observe a similar effect in the crossing behavior of pedestrians in terms of crossing predictions. This could be due to our strategy of priming participants before the experiment by informing them about the different characteristics of AVs and HDVs that AVs would always yield to them to prevent a collision and HDVs may or may not yield to them. We have done this to approximate pedestrian behavior once they are accustomed to conflict-avoidant AVs after long-term exposure in the future. Hence, the difference between our results and those of Kalatian and Farooq (2021) might indicate differences in the novel and primed mental models of pedestrians when they encounter AVs. Furthermore, Kalatian and Farooq (2021) reported that some teenage participants performed deviant behavior against virtual vehicles once they realized that vehicles react according to their crossing behavior. Participants then would play with them by moving back and forth on the street. The authors pointed out future implications of deviant behavior toward AVs in their work, and their statements are in line with our general crossing results and the study of Moore et al. (2020) in this regard.

Moreover, Colley et al. (2022) tested pedestrian behavior in the presence of constant oncoming AVs, which would not yield for participants. Their results showed that after a couple of passing AVs, pedestrians relied on the prior information of an emergency braking system of AVs and preferred crossing for saving time. However, they have only tested this condition for AVs. In our experiment, we utilized always yielding AVs and yielding and non-yielding HDVs. To draw a clearer picture of whether pedestrians treat AVs and HDVs differently, a follow-up study including non-yielding HDVs and non-yielding AVs can support our risk-controlled results from another perspective.

The gamification of our experiment further enabled us to manipulate conditions that directly affect individual gains in the form of earning points and earning extra reimbursement in euros. Task urgency was directly linked to maximizing the incentive participants would gain. Generally, we found urgent scenarios to predict higher chances of crossing instead of waiting, confirming that participants showed more deviant behavior under time pressure, in line with Morrongiello et al. (2015), Schneider et al. (2019), and our theoretical expectations formulated in H1.

Results of our analysis also indicate that different forms of social control, indeed, influence individuals’ decisions to jaywalk. We find the mere presence of cues signaling formal norm enforcement (police presence) to deter individuals from crossing, hence confirming H3c. This finding is likewise corroborated by participants’ responses: participants state that police played a role in the majority of their decisions. In this condition, our approach and application of formal traffic norm cues differ from the work of Jayaraman et al. (2019) in essence. As Jayaraman et al. utilized signalized and non-signalized pedestrian crossings as a factor for investigating the effect of formal traffic rules on pedestrians’ crossing decisions, we have placed the police officer character as a mere cue for the presence of legal authority. Moreover, this character did not have a definite effect on traffic rules as in the case of a traffic light that Jayaraman et al. used. In our experiment, jaywalking was not illegal and police presence did not directly signify a punishment if participants jaywalked. Moreover, 50% of the time, the police were not effective in the trials. Another difference in our approach from Jayaraman et al. is that we tested for deviant behavior of pedestrians in the presence of legal authority, whereas they tested for pedestrian trust in automated vehicles in the presence or absence of a formal traffic sign. Our results are also in line with Camara and Fox (2020). They suggested that rare large penalties could be replaced with milder and more frequent negative utilities, hence preventing pedestrians from acting deviant. In our study, the mere cue of legal norms without certainty of sanctioning seemed to deter our participants from crossing.

Looking at the effect of negative social cues, that is, the effect of cues signaling low levels of social conformity, we see a strong increase in deviant behavior with a crossing probability up to 100%. These results match with the results of Colley et al. (2022) and the reporting of Faria et al. (2010), where they observed an increase in crossing behavior probability when other pedestrians started to cross. As this finding indicates the negative effect of cues signaling low levels of norm compliance on deviant behavior of participants, this strong effect might also result from our experimental design. Compared to a mere cue, our implementation of the negative bystander effect stopped the oncoming traffic, thereby transforming the individual decision to jaywalk into a decision to free-ride. Moreover, Mahadevan et al. (2019) reported an insignificant effect of crossing group behavior on participants’ crossing decisions on their pedestrian simulator, which is opposite to our findings. Hence, we cautiously confirm our hypothesis H3b, and overall, negative social cues are worth deeper research.

In our experiment, positive social cues represented the social sanctioning in the forms of a mother and a child character. We did not observe a difference in crossing behavior predictions in this condition when compared to being alone in the scene. As a result, we failed to confirm H3a. However, when we explicitly asked participants how their behavior would differ in real traffic situations, the majority stated that they would generally abide by the rules in the presence of children and police. Overall, this seems indicative that even though participants were in a low-fidelity virtual environment with a delivery task assigned to them, they were affected by the social control of bystanders. However, social sanctioning might play a bigger role in real-life interactions than in the virtual environment.

When we explored the potential interaction effects of vehicle type by task urgency or social control on crossing predictions, we have only found a significant difference between AVs and HDVs in the mother and child condition compared to being alone. This effect existed only in risk-controlled trials, meaning that when the risk of collision is balanced, having the mother and child in the scene decreased the crossing probability in front of HDVs, whereas it did not change the crossing probability in front of AVs. A potential explanation might be that when mother and child existed in the scene, participants were more risk-avoidant and cautious about crossing in front of HDV, whereas they still relied on the defensive nature of AVs, and they did not alter their behavior in the presence of the mother and the child. On the whole, to our knowledge, no study regarding pedestrian–AV interaction considered the effect of social norms by focusing on the effect of bystanders as we utilized.

In conclusion, it seems that AVs of the future will be the inferior counterpart of interaction with humans if they remain risk aversive and if there is an imbalanced distribution of crash risk among human-driven and automated vehicles. When the costs of deviant behavior are balanced while crossing in front of these vehicles, the sole effect of automation attributes does not influence the crossing behavior, which supports the idea that, in essence, people would treat the AVs the same as HDVs if they behave similarly. As the defensive nature of AVs is essential for the safety of future mixed traffic and for the acceptance of AVs, this might incentivize individuals to exploit them in the long term. Lastly, our exploration of social norm dynamics reveals that social control, especially legal cues, carries the potential of being the regulator of humans’ deviant behavior.

### 5.1 Limitations and Future Directions

We used the gamification approach to eliminate task fatigue in the experiment and make the participants more involved with the task. Most participants seemed to enjoy the idea of earning points. Furthermore, the point system helped us establish costs and benefits in a more realistic way than leaving these concepts to participants’ imaginations in our VR study. We have observed that gamification fitted well with repetitive tasks because it had placed these tasks conceptually in a meaningful context. However, because we used gamification, we took the liberty of keeping the environment in low fidelity. The effect of this decision was reflected in the experienced realism ratings of participants in IPQ results. Benefiting from a more realistic environment in the next iteration can improve experienced realism, hence an overall more immersive experience, which might provide for more fine-grained results.

Because we primed our participants that AVs would always be conflict-avoidant and yield to them, we did not include non-yielding AVs in our design. A future study where we introduce non-yielding AVs can help us to position our current results regarding risk control in a more validated place.

We had a rather young sample with individuals from similar educational backgrounds. Deb et al. (2017b) reported, in their PRQF scale validation study, that younger people were more receptive toward AVs. We could confirm this finding with our young sample. However, a more diversified sample could draw a more realistic picture of the existing traffic dynamics. Moreover, we arranged the traffic flow unidirectional in our experiment to keep the task less complicated and make sure that participants would not miss the target vehicle. However, this can be enhanced with some alterations in the study design. Furthermore, we have given participants the repetitive task of crossing the same street. Even though we have emphasized the pizza delivery task in our instructions, and on our game concept, benefiting from different virtual streets could have blinded our manipulations even better.

## Data Availability

The original contributions presented in the study are included in the article/Supplementary Material. Further inquiries can be directed to the corresponding author.

## Appendix A List of Open Questions.

• What have you paid attention to when you were playing the game?
• Did police or other pedestrians affect your crossing decisions? How?
• Did timers or urgency symbols affect your crossing decisions?
• Did vehicle types influence your crossing decisions?
• How would your street-crossing behavior differ in real-life situations?
       -When you see people who wait for the cars to go first.
       -When you see people who do not wait for the cars to go first.
       -When you see children around.
       -When you see police around.
     -When you are in a hurry.
